# Suicidal Career in Severe Depression among Long-Term Survivors: In a Followup after 37–53 Years Suicide Attempts Appeared to End Long before Depression

**DOI:** 10.1155/2013/610245

**Published:** 2013-12-23

**Authors:** Lisa Crona, Alexander Mossberg, Louise Brådvik

**Affiliations:** Department of Clinical Sciences, Division of Psychiatry, Lund University Hospital, 221 85 Lund, Sweden

## Abstract

*Objective*. To describe the suicidal career in the long-term course of severe depression. *Subjects and Method*. Seventy-five former in-patients were interviewed by telephone about course of depression and suicide attempts 37–53 years after index admission. Medical records were read in many cases. *Results*. 29 subjects had attempted suicide, 13 repeated, 10 made severe, and 13 violent attempts. The risk of suicide attempt decreased by 10% for every decade spent depressed. Suicide attempts were made early in course of depression, and more time was spent depressed after suicide attempts than before. *Conclusions*. A healing process of the suicidal career, which may occur long before the end of the last depressive episode (sometimes decades), is proposed.

## 1. Introduction

Depression is a common disease [1]. It is one of the leading causes of disability worldwide, and it is a major contributor to the global burden of disease [2]. Suicide is a feared outcome of depressive disorders. A review article calculated the life time risk of committing suicide to 6% in affective disorder [3]. Mood disorders is one of the diagnoses that convey the highest risk for suicidal behavior in high-income countries [4]. Different studies have shown that 43% to 60% of all suicide victims suffered from mood disorders [5–8], and, when dysthymia was included, 90% of the suicide victims were shown to have suffered from a depressive disorder according to one study [9]. Severe depression (major depressive disorder with melancholic or psychotic features/endogenous depression) has been shown to predominate in the depressive group of suicide victims [10, 11].

Attempted suicide is important in the study of suicide since it is the most important known risk factors for completed suicide [12–15]. Different studies have shown rates of attempted suicide between 15% [13] and 34% [16] for patients with major depressive disorder. Subjects with major depressive disorder who have attempted suicide have an increased risk of reattempting [17].

The risk of suicide attempts among depressive patients has been found to be strongly associated with the presence and severity of depressive symptoms [17]. It has also been proposed that length of time spent depressed is likely to be the major risk factor for the overall long-term risk of suicide attempts [13]. Number of depressive episodes has been shown to be positively correlated with the number of suicide attempts [12, 18]. On the other hand, it has been proposed that suicide attempts occur early in the course of depression [19–23] and it has been shown that those who have attempted suicide are likely to repeat attempts soon after the first attempt [14, 24].

Thus, the knowledge about suicide attempts during the course of depression is contradictory; longer time spent depressed increases the risk of attempt, still attempts seem to occur early in the depressive course. We have previously shown that the very long-term course of severe depression can be described according to six different long-term courses. Both recovery and remission were possible in the late course [25]. To the best of our knowledge, however, no studies have related suicide attempts to the very late course of severe depression.

The aim of this present study was to study the suicidal career among these long-term survivors of severe depression. First, the occurrence of suicide attempts and reattempts were noted, and then severity and violence of the attempts were evaluated. Second, the risk of suicide attempt during the course of depression was investigated. A connection between the suicidal career and the depressive course clusters was studied. Finally, did the subjects stay depressed after suicide attempts without making more attempts?

## 2. Subjects and Methods

### 2.1. Subjects

The subjects in the present study are a sample previously described according to their long-term course of severe depression [25]. Between 1956 and 1969, a multidimensional schedule, including diagnoses, was used to rate all in-patients at the Department of Psychiatry, University Hospital Lund, Sweden [26]. The ratings were made at discharge of the patients and were performed by senior psychiatrists with at least three years of training. In all, 1206 subjects were diagnosed with severe depression/melancholia during that time span. Of these there were 700 women and 506 men. According to one study [27], including 178 of these patients, at least 91% of the patients met the criteria for severe depression with melancholic or psychotic features according to DSM IV [28], and the actual number was probably higher, as symptoms may be underreported in the records.

Out of the 1206 subjects, 471 were born in 1920 and later and could therefore be expected to be alive and able to participate in a third followup 2006–2010. They were followed up by the Central Bureau of Statistics Sweden until 1 May 2006. After excluding those who had died (*n* = 196), those who could not be traced (*n* = 106), and those who were contacted but were not able to participate due to dementia, other illness, or not remembering ever being depressed, 150 remained. Of these 75 agreed to join the study (51 women and 24 men) and were interviewed by telephone [25].

### 2.2. Methods

The interviews were based on a structured life-chart procedure, as proposed by Roy-Byrne et al. [19]. The interview consisted of questions about social situation, occupation, physical illness, current medication, course of depression, cause of onset and cause of recovery/remission, treatment, suicide attempts, childhood, alcohol, and so forth. Permission to read their records was given by 44 subjects. Thirty-six records were found and read.

A cluster analysis has previously been performed and six course clusters were identified describing the course of depression [25]. In summary, there was a short-term course with or without recurrence or a chronic course with or without late remission.

In the present study the suicide attempters and the nonattempters of the sample followed up in 2006–2010 were compared regarding their long-term course of depression. Parameters considered for comparison were gender, debut age of depression, time of depressive episodes, the course of depression previously described, time of suicide attempts, number of attempts, severity of attempts, and whether a violent method had been used in the attempts. The year a subject first was admitted due to depression was defined as debut year of severe depression, usually the first episode. The information on suicide attempts was obtained from telephone interviews and records, when possible, (*n* = 22) or from the interviews only (*n* = 7).

Suicide attempt was first scored by severity on the basis of the schedules introduced by Motto [29] and Weisman and Worden [30]. We used a rather broad definition of self-harm, including what Motto called suicidal gestures, in cases where intent was difficult to determine on the basis of case records. The original study started in 1984 and the same definitions were used in the two followups in 1998 and 2006. Some more recent investigators also used a broad definition of self-harm without considering the degree of intent [31–33], which would include suicidal gestures and probably some aborted attempts (serious intent but interrupted by the subject before self-harm was caused), which were also categorized if possible. The latter have been described by Marzuk et al. [34] and have been associated with actual suicide attempts [35].

The rater (AM) was blind to the history of the subjects and evaluated severity and violence according to the information about the individual attempts found in medical records and documentation from interviews. Severity of suicide attempts was graded according to previous measures [15] with special reference to severe suicide attempts: either highly lethal suicide attempts, requiring intensive care, which were considered medically severe and/or if there were precautions against discovery and strong regret at failure to die, which were considered psychologically severe (the latter was not found in the present study).

Grading of severe attempt was possible in 27 out of 29 subjects. In another case we knew that there had been definite suicide attempts, but there were also later attempts for which we had insufficient information. Thus we could conclude that there had been definite attempts but not exclude severe attempts in that case, and grading for definite attempts was possible for 28 persons.

Suicide attempts were also defined as violent or nonviolent, which was possible in 27 out of 29 subjects. Superficial cutting and drug intoxication were defined as nonviolent methods and all others were defined as violent [36].

For one subject the exact time for the one and only suicide attempt was not possible to find out. For another the time for the last attempt was known but not the exact time for the earlier attempts, and therefore the length of suicidal career could not be calculated for this subject. Thus, the time of the suicidal career could be estimated in 27 cases.

### 2.3. Statistics

A poisson regression was used for modelling how the instensity of suicide attempt per person and year varied by time.

Wilcoxon rank sum test was used for comparison of time spent depressed before and after suicidal career.

### 2.4. Ethic Approval

The study was approved by Lund Medical Ethics Committee 2003.

## 3. Results

### 3.1. Occurrence and Characteristics of Suicide Attempts

Out of the 75 subjects, there were 29 (39%) who had attempted suicide, 8 men and 21 women. Reattempts were made by 13 (45%) of the attempters. One subject stands out, having made twelve attempts. Altogether the 29 suicide attempters had made 67 attempts (Table 1).

The characteristics of suicide attempts are presented in Table 2. In all, 10 out of 27 subjects (37%) had made at least one severe attempt and another 12 had made at least one definite attempt; in all 22/28 (79%) had made at least one definite or severe attempt, that is, 22/75 (29%) of the total sample. Thirteen of 27 subjects (48%) had used a violent method in at least one suicide attempt.

Thus occurrence of suicide attempts as well as repetition, severity, and use of violent method was not uncommon among long-term survivors of severe depression.

### 3.2. Course of Depression and Suicide Attempts

**Figure 1:**
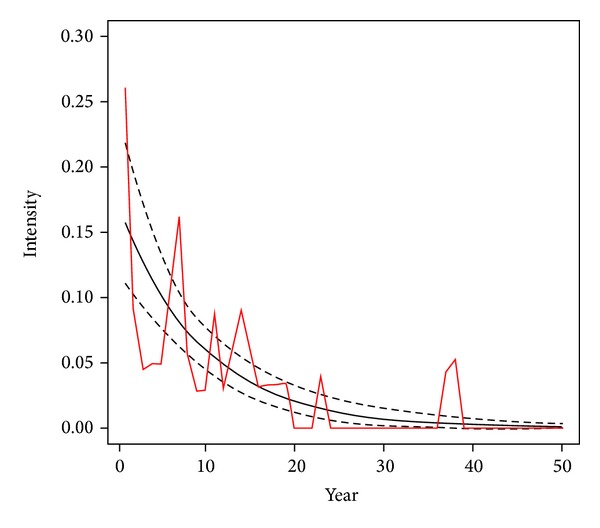
Intensity of sucide attempts during course of depression by years.

The distribution of suicide attempts during the course of depression is presented in Figure 1.

A poisson modeling showed that the intensity of suicide attempt actually decreased by 10% per decade (incidence rate: 0.90, CI = (0.87, 0.93), and *P* value <0.001).

When comparing the occurrence of suicide attempts to the course cluster of depression, we found a nonsignificant trend towards a higher risk in chronic course. There were 16/51 (31%) suicide attempters in the group with short-term course with or without recurrence and 13/24 (54%) in the group with chronic course with or without late remission (Fisher's exact test = 0.077). There were somewhat higher rates of repetition, 8/16 (50%), in the first group compared with 5/13 (38%) in the chronic group. Thus the risk of suicide attempt tended to be higher in the chronic group, but there was no increased risk of repetition.

### 3.3. The Suicidal Career among Attempters

**Figure 2:**
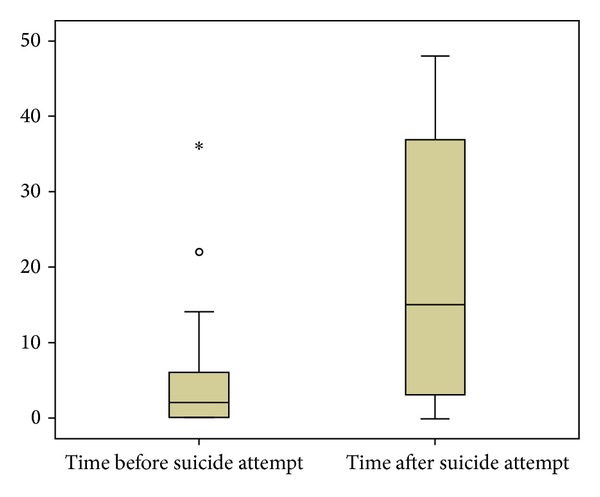
Box plot of years spent in depression before and after first and/or last suicide attempt.

The suicide attempts among those who attempted suicide related to course of depression are presented in Figure 2. Suicide attempts were made early in the course of depression. The time from debut of depression to first suicide attempt was significantly shorter than the time after the last suicide attempt to the end of the last depressive episode (Wilcoxon *z* = 3.493,  *P* < 0.001). Thirteen of 27 suicide attempters made their first attempt within one year from debut of first depressive episode. Mean time from first depressive episode was 5.30 years (±8.33) and median 2 years. Mean number of years from last suicide attempt to recovery or to telephone interview (when still depressed) was 18.96 years (±16.87) and median 13.5 years.

Only five out of twenty-nine subjects had a late debut (more than a decade) of suicide attempts with 12, 13, 14, 22, and 36 years, respectively, between first depressive episode and first suicide attempt. However, the person who first made an attempt after 36 years made their two only attempts during a late relapse of depression, and the other outlier making an attempt after 22 years did it late in the course of chronic depression.

Fifteen of the suicide attempters experienced depressive episodes for more than ten years after their last attempt, out of which ten subjects had continuous depressive episodes for 30 years or more without making any more attempts.

Among those who made repeated suicide attempts, the attempts were usually made within a short time span; 6 out of 13 had a suicidal career lasting for one year or less and 11 within 5 years. Only two persons had a much longer time from first to last attempt, lasting for 13 or 17 years (the first made continuous attempts and the other made a second attempt late in course; both had continuous depression after their last suicide attempt). Mean debut age of depression in the whole sample was 27.25 years [25], and for the subjects making suicide attempts it was 24.9 years. Mean debut age of suicide attempts was 30.2 years (median 28).

Thus the suicidal career came to an end long before the end of depressive episodes, and there was usually no relapse of suicide attempts later in course.

### 3.4. Treatment

In the case records available, the rates of adequate treatment for the depressive episodes in the long-term course of depression did not differentiate between those who made suicide attempts and those who did not (77% versus 77%). There was no difference in rates of treatment at time of the interview (31% versus 29%).

## 4. Discussion

### 4.1. Main Findings

Suicide attempts were not uncommon among long-term survivors of severe depression, and neither were repeated, severe, or violent attempts. In the present study the risk of suicide attempts decreased by 10% for every decade of depression. The subjects in this study attempted suicide early in their course of depression and debut attempts were often made while subjects were young. Furthermore, suicide attempts occurred only for a limited period of time among repeaters; 11 of 13 who made repeated attempts had a suicidal career lasting for five years or less. Finally, almost all suicide attempters continued to have depressive episodes for many years and they spent significantly more time depressed after their last suicide attempt than before it.

These findings are in concordance with other studies, in which suicide attempts have been found to occur early in the course of depression [20–23, 37] and reattempts follow soon after suicide attempts [24]. However, the present study contradicts the findings that time spent depressed increases the risk of making new suicide attempts [9, 10, 17, 18]. In a previous study on 100 suicide victims and matched controls selected from the same original sample of 1206 former in-patients with a severe depression/melancholia, it was found that those who continued to make suicide attempts throughout their course of depressive episodes were more likely to finally commit suicide [38]. The increased rates of suicide attempts among suicide victims may be seen as an indication of an end of the suicidal career among survivors. However, in that previous study the median followup was only 12.8 years in the suicide group, as the case records of the controls were cut, when the suicide victim they matched died. Moreover, the length of time of depression after last suicide attempt was not measured. Risk of late relapse of suicide attempts could not be concluded either.

In the present study the number of severe and violent attempts was rather high; 37% and 48% respectively, of the subjects had used a severe and/or violent method for at least one attempt. Less severe and less violent methods among suicide attempters have been proposed in other studies [39, 40]. On the other hand, it has been shown that subjects who make severe suicide attempts and those who complete suicide have much in common [41], and violent suicide attempt has been related to completed suicide [42, 43]. Thus, it is noteworthy that a high proportion of long-term survivors of severe depression with melancholic and/or psychotic features made severe/violent suicide attempts but never completed suicide. The rates of repetition were somewhat higher compared to other previous investigations, 45% versus 25% in another study, in which frequent repetition in females predicted suicide [44], but similar to another one, where half of the patients made repeated attempts [45].

In comparing the course of depression, we found a trend towards a higher risk of suicide attempt among those who had a chronic course of depression, though suicide attempts generally occurred before the depression was chronic and there did not seem to be a high risk of repetition of attempts during the chronic course. There may be some risk inherent in depression as such rather than a risk of continuous course in chronic cases, but the sample is too small to allow conclusions. Further research is needed.

Treatment did not seem to differentiate between those who had made suicide attempts and those who had not, but certain data were difficult to receive in retrospect during this long time span. We found that some of formerly depressive patients had recurrent depressive episodes *with* or *without *antidepressant treatment. Some made suicide attempts during treatment and some without treatment. The suicidal career may end despite recurrent depressive episodes as a natural course of depression with suicidal behavior in untreated cases. It is also possible that some stop making suicide attempts during later depressive episodes despite antidepressant therapy. In these cases it cannot be excluded that antidepressant therapy has *no* effect on depression but on suicidal behavior *only*. This possibility cannot be concluded either, as we do not know with certainty that antidepressants are the cause of the end of suicidal behavior, when it has not had an effect on depression.

We propose that the suicidal career may end despite severe, violent, or repeated suicide attempts and also ends long before the recovery of depression (sometimes decades), usually without relapse of suicide attempt. Recovery from depression does not seem to be a precondition for ending of a suicidal career and continuous depressive episodes do not seem to increase the risk of suicide attempts.

### 4.2. Strengths and Limitations

The material in this study is unique since the subjects have been followed up during a very long period of time, between 37 and 53 years. To the best of our knowledge no one has described such a long course before. This enables the study of a very long-term perspective of the course of depression and how it affects the career of suicidal attempts. It is also a limitation, since the subjects get highly selected. From the original 471 subjects 169 were alive and possible to track. That some subjects could not be traced was sometimes due to the fact that some had old civic registration numbers without the four last digits, some had emigrated, and some did not have the correct telephone number in the registers. This fact did not probably contribute to an important selection bias. Out of 150 subjects who were able to participate 75 were included in the present study. However, the goal of the study was to look at the long-term survivors of severe depression. The deceased subjects may well be compatible with those being alive when it comes to suicide attempts, since they have died from other causes than suicide. The prevalence of suicide attempts in the sample is somewhat higher compared with those found in other studies [12, 13, 16]. However, it is similar to the rates among other survivors from the same sample; 38% versus 34% [38]. Hence, the present sample is probably representative for the larger sample of patients with severe depression/melancholia despite of the limited participation in the present study. The small total number of suicide attempts, independent of representativity, is another limitation and the findings need replication. However, we could, with greater certainty, conclude that there is not an *increased* risk of suicide attempts during course of depression despite the small sampling size.

In a retrospective study you always have to deal with the limitation of recall bias. The length of the study and the fact that the subjects were old may worsen this problem. When the course of depression was investigated during a previous study [25], the telephone interview records were compared with medical records. It showed that subjects in general had estimated more depressive episodes than there were episodes in their records—no underestimation seemed probable. It may also be assumed that a suicide attempt is less easily to be forgotten than a depressive episode.

## 5. Conclusions

According to our findings, the long-term survivors of severe depression in our study tend to make single or repeated suicide attempts during early course of depression and may later continue to have depressive episodes for many years, sometimes decades, without making any more attempts. The attempts can be severe and violent or repeated but still not lead to a longer suicidal career or to completed suicide. A healing process of the suicidal career, which occurs long before the healing of depression, is proposed.

## Figures and Tables

**Table 1:** Distribution of number of attempts by repetition.

Number of attempts *N* = 67	Number of subjects (men) *N* = 29
1	16 (5)
2	5 (0)
3	3 (1)
4	2 (1)
5	1 (0)
7	1 (0)
12	1 (1)

**Table 2:** Characteristics of suicide attempts: severity and violence related to repetition by individuals (women + men).

	Severe attempt	Violent attempt	Severe and violent	Severe and or violent	Nonsevere/violent	Total
Single attempt	3 (1 + 2)	6 (4 + 2)	2 (0 + 2)	7 (5 + 2)	8 (5 + 3)	16 (11 + 5)*
Repeated attempts	7 (6 + 1)	7 (6 + 1)	5 (4 + 1)	9 (8 + 1)	3 (2 + 1)	13 (10 + 3)*

Total	10 (7 + 3)	13 (10 + 3)	7 (4 + 3)	16 (13 + 3)	11 (7 + 4)	29*

*In two cases method and severity could not be concluded; hence the horizontal sum does not fit.

Severe and violent may refer to two different attempts. Two persons made 2 severe attempts and 3 persons made 2, 3, and 4 violent attempts, respectively.
